# Pests or prey? Micromammal species within an ancient anthropic environment at the Norse settlement site of Tuquoy (Westray, Orkney)

**DOI:** 10.1098/rsos.221462

**Published:** 2023-04-05

**Authors:** Andrzej A. Romaniuk, Lore G. Troalen, Robin Bendrey, Jeremy S. Herman, Olwyn Owen, Catherine Smith

**Affiliations:** ^1^ School of History, Classics and Archaeology, The University of Edinburgh, Teviot Place, Edinburgh EH8 9AG, UK; ^2^ Institute for Advanced Studies in the Humanities, The University of Edinburgh, Hope Park Square, Edinburgh EH8 9NW, UK; ^3^ Department of Collections Services, National Museums Scotland, Chambers Street, Edinburgh EH1 1JF, UK; ^4^ Department of Natural Sciences, National Museums Scotland, Chambers Street, Edinburgh EH1 1JF, UK; ^5^ Institute of Archaeology, University of the Highlands and Islands, Orkney College, East Road, Kirkwall, Orkney KW15 1LX; ^6^ Alder Archaeology Ltd, 55 South Methven Street, Perth PH1 5NX, UK

**Keywords:** micromammal, predation, commensalism, archaeology, introductions, Orkney

## Abstract

Micromammals, like rodents and shrews, adapt rapidly to take advantage of new food sources, habitats and ecological niches, frequently thriving in anthropogenic environments. Their remains, often retrieved during archaeological investigations, can be a valuable source of information about the past environmental conditions as well as interspecies interactions and human activity. However, the research on such finds rarely covers multiple approaches, often relying on single species or data type (e.g. identification/information for proxy studies). Here we investigate micromammal remains from the Norse and medieval (AD tenth–fourteenth centuries) archaeological site at Tuquoy, Orkney, to elucidate the relationships between micromammals, humans and other species present using a variety of data. Four micromammal species were identified, and their species dynamics as well as relationships with humans could be inferred by tracking changes in spatial and temporal location of remains, from their taphonomic history and by age estimation for individual animals. A larger, predatory assemblage was also identified, with species composition differing from that in the rest of the archaeological assemblage, and possibly therefore representing small mammal species composition in the wild. The assemblage was probably deposited by a diurnal raptor, though identification to species is not certain due to post-depositional processes.

## 1. Introduction

The composition of the Orkney Islands' (Scotland, UK) micromammal fauna, such as rodents and shrews, has been strongly influenced by their relationship with humans. The post-glacial land connection between the Orkney archipelago and mainland Britain disappeared too early for natural species migration to take place [1]. All terrestrial mammals, and many other faunal and floral species, were most likely introduced by humans since the time of the Neolithic intensification of maritime contacts [2–9]. One of the most studied Orcadian mammals is the ‘Orkney vole’, an introduced population of the common vole *Microtus arvalis*, which is widespread in mainland Europe but otherwise absent from the British Isles [10]. Molecular phylogenetic studies pointed towards its introduction from the western European mainland around the fourth millennium BC, possibly as a single human-mediated event [5,7,11–13]. Previous research on archaeological material from Skara Brae, both micromammal remains [9,14] and coprolites [15], revealed large quantities of vole remains in refuse deposits as well as individual vole bones within the faeces of dogs, perhaps showing the outcome of pest control methods or even intentional collecting of those animals by Neolithic Orcadians.

However, beyond typically commensal species, like house mice *Mus* spp*.* [16–20] or black rats *Rattus rattus* [21–23], micromammals are an under-researched subject in archaeology. On Orkney the history and population dynamics of microfauna beyond voles is vaguely known, with the majority of knowledge coming from nineteenth- and twentieth-century observations [1] and evolutionary research on modern populations [24–26]. Due to issues with handling such small material [27], and micromammal retrieval often a by-product of sampling for other small finds, micromammal archaeology rarely goes beyond simple species identification or non-archaeological investigation of a small part of the data that are potentially available.

From the perspective of quaternary science, micromammal remains are a valuable source of information and should be treated as such also in archaeology. Micromammals are an important part of every ecosystem they inhabit, providing food for many different predators, including numerous bird and mammal species [28], as well as forming complex relationships with humans and other animals [29,30]. Due to their short lifespan and high reproduction rate, micromammals react rapidly to both environmental [31] and human-mediated changes [11], and therefore make a good proxy for natural and man-made processes. Moreover, shifts in micromammal taxonomic composition or population dynamics may affect their habitat and other species present there. This is especially obvious in the case of islands where new species have been introduced [32,33]. For a long time, micromammal finds were treated as a proxy for taphonomical research on predation [28,34–36], resulting in a well-developed quantitative methodology applicable to archaeological finds.

Work on the micromammal remains from Tuquoy, carried out in 2017–2019 as part of a wider PhD project [37], provided a unique opportunity to examine micromammal population dynamics at this archaeological settlement before and during its construction, utilization and later abandonment. The aim of the Tuquoy research was to demonstrate the methodological validity of researching micromammals in an archaeological setting through using a wide set of approaches, and to provide the basis for further micromammal research in Orkney archaeology. It was achieved by examining all the micromammal materials sampled from the site, quantifying data required for both taphonomic analysis and reconstruction of animal mortality profiles. Emphasis was put on establishing the sequence of species arriving at the site, finding any difference between the overall micromammal population within the site and the external fauna, as well as evaluating possible predation within or around the man-made structures.

## 2. Materials and methods

### 2.1. Materials

**Figure 1.**
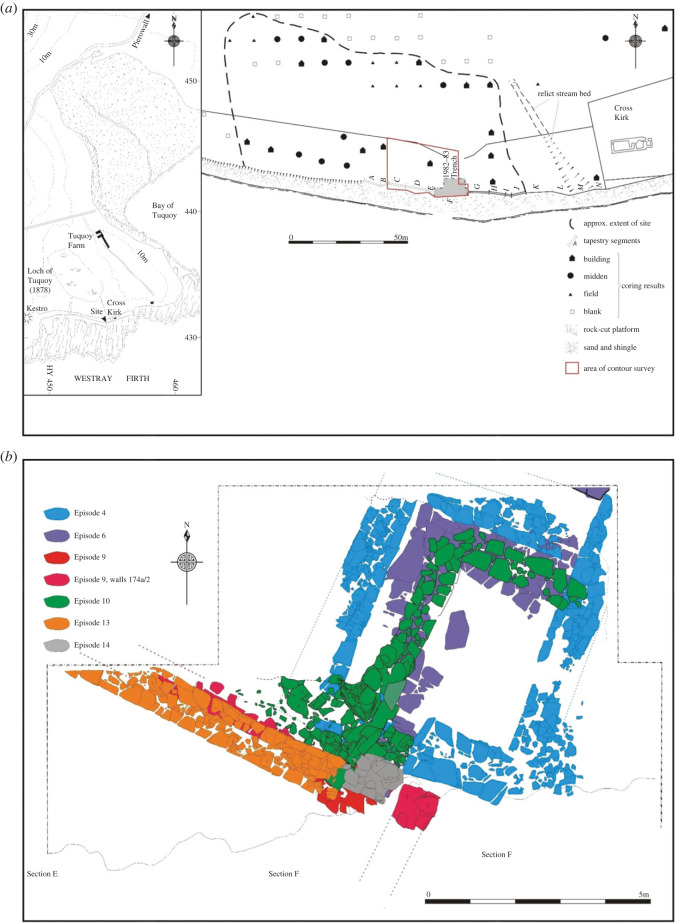
Tuquoy. (*a*) Map showing the location and surviving extent of the site and (*b*) plan of the major structures investigated in the 1982–1983 excavation area. In (*b*), two phases are represented: Phase 3 (Episodes 4 and 6: construction and refurbishment of the hall) and Phase 4 (Episodes 9, 10, 13 and 14: construction and rebuilds of the workshop). © Historic Environment Scotland.

The archaeological site at Tuquoy is a late Norse and medieval settlement located adjacent to, and associated with, the AD twelfth-century chapel of Cross Kirk, in Westray, Orkney [38] (figure 1). The presence of a chapel reflects the high status of the settlement during that time and it was probably the local ruler's seat. The site was investigated over several excavation seasons, in 1982–1983, 1988 and 1993. The trial work concentrated on an area less than 100 m^2^, containing the remains of a Norse ‘hall’ and a substantial ‘workshop’ and the terrain around them, including waterlogged deposits discovered on the beach nearby, at the base of a low cliff. Natural deposits and early activity before the hall was constructed, as well as abandonment layers and later natural and anthropogenic contexts, were also documented. Due to technological and time restrictions, usually only one bucket (about 10 l) was collected from each archaeological context for sieving by 1 mm mesh during the first excavation season. During the second season, the combination of 4 mm followed by 0.5 mm mesh was used, with many contexts sieved in full due to a narrower area of excavation. A few clay contexts could not be properly sieved and only hand-picked remains could be retrieved from these. The recording structure of the site stratigraphy, used in this paper and related appendices in the electronic supplementary material, is as follows:

Single context → Block (contextually, spatially and temporarily similar group of contexts) → Episode (group of blocks, representing short time period within the larger phase) → Phase (longer time period, visible throughout the site).

While there is no final publication of the site available yet, unpublished data including the stratigraphic report, guidelines for specialists working on Tuquoy environmental material, radiocarbon dates and zooarchaeological reports were provided to the authors by the team currently working on a site-specific monograph [39] (S Hamilton-Dyer, A Krus, J Rackham October 2017–June 2018, personal communication).

### 2.2. Methods

All samples with small skeletal remains were sorted to separate micromammal elements from the rest of the finds. Anatomical and taxonomic identification of these remains relied on reference material from the National Museums of Scotland vertebrate collection, together with published descriptions [40–43] and unpublished reference charts. Specimens that could not be identified to species, such as smaller bones (vertebrae, ribs, metapodials, phalanges, calcanei, tali and isolated individual cranial bones) or significantly altered remains, were recorded only as ‘unidentified rodent’ (similar to the vole/mouse category in [6]). Murid postcranial bones could be distinguished from arvicolid or other unidentified bones, but rarely could be associated with specific species, and were thus recorded as ‘unidentified mouse’.

Methods for quantitative and taphonomic analysis were based on those of Andrews [28], together with more recent studies [9,14,34,35] and considering their applicability to the archaeological setting [36]. For quantitative analysis, the number of identifiable specimens (NISP) was established for each sample, counted individually for key anatomical elements (mandibles, maxillae, scapulae, humeri, radii, ulnae, pelves, femora, tibiae, vertebrae, metacarpals, phalanges, calcanei, tali, ribs as well as loose incisors and molars) and jointly for other bones. The minimum number of individuals (MNI) was established for sample and context level and each species class present. MNI estimation was based on NISP results while taking into account differences in discoloration or different molar wear. From Andrews' work [28], a number of quantification methods using NISP and MNI were incorporated into this study, including relative abundances of key elements, average relative abundances, and indices expressing differences between different parts of anatomy (postcranial to cranial elements, distal to proximal limb bones) and how the number of isolated teeth relates to remains with missing teeth (percentage of isolated teeth). Details, including how the equations work, are in table 1.

**Table 1.** Summary of equations used in this study, taken from Andrews ([28, pp. 45–90] and table 3.2 and 3.9–11 in [28]).

equation (X 100%)	full term	explanation	elements considered
Ri = NISPi / (MNI × Ei)	relative abundance	ratio of a specific element NISP (NISPi) to expected anatomical elements numbers (MNI multiplied by a number of elements in a typical skeleton, Ei)	BONES: (Ei = 2) maxilla, mandible, scapula, pelvis, humerus, ulna, radius, femur, tibia, calcaneus, talus, (Ei = 36) vertebra, (Ei = 20) metapodial, (Ei = 56) phalange; LOOSE TEETH: (Ei = 12) molars, (Ei = 4) incisors
$\bar{R}=$ΣRi/nR	average relative abundances	ratio of the sum of relative abundances (ΣRi) to the number of relative abundances present (nR)	as in relative abundances, either all or excluding loose teeth
pc × 8 / c × 5	full index of postcranial (pc) to cranial (c) elements		pc = NISP(femurs + humeri + tibiae + radii + ulnae); c = NISP(mandibles + maxillae + isolated molars)
pc / c	simple index of postcranial (pc) to cranial (c) elements		pc = NISP(femurs + humeri); c = NISP(mandibles + maxillae)
dist / prox	index of distal (dist) to proximal (prox) limb elements		dist = NISP(tibiae + radii); prox = NISP(femurs + humeri)
T(isolated) / T(missing)	isolated incisors/molars	index of isolated teeth against empty alveolar spaces (i.e. missing teeth); separately for molars and incisors	T(isolated) = NISP(isolated teeth); T(missing) = Count of empty alveolar spaces found
T(broken) / T(all)	broken incisors/molars	index of broken teeth against all teeth found; separately for molars and incisors	T(broken) = NISP(broken isolated teeth) + Count of broken teeth *in situ*; T(all) = NISP(isolated teeth) + Count of all teeth *in situ*

For taphonomic analysis, cranial, long bone and tooth breakage (also named fragmentation) were recorded. Bone breakage was scored in a manner firstly explained in Andrews [28], following more exclusive classes for cranial and mandibular breakage present in more recent research [37,44]. Cranial breakage included four stages, from complete skulls to isolated bones or their fragments. Mandibular breakage also was divided into four stages, from complete bones to fragments missing the ascending ramus, with the inferior edge broken. Long bone breakage data for detailed analysis followed Andrews' [28] example, with scoring including complete bones or fragments of specific element region (proximal epiphysis, distal epiphysis, shaft). Some fragments were scored to more than one category. For data more aligned with NISP counts, in the electronic supplementary material, appendices, the authors also included simplified complete/broken dichotomy for postcranial elements.

Evidence for digestion was also investigated. For detailed analysis, digestion marks on teeth were scored according to Andrew's methodology, from 1 (rounding on the teeth tips) up to 5 (extreme digestion, often with changes perforating the tooth [28,35,36]). For the appendices, tooth digestion was counted only as present, similarly to humeral/femoral epiphyses digestion. High-magnification micrographs for detailed taphonomic studies were obtained with a BSC 20.00 kV scanning electron microscope (CamScan MX2500).

Ageing of micromammal remains was based on epiphyseal long bone fusion for both murids and voles and on molar tooth wear for murids only. Epiphyseal fusion was divided into three categories and modified according to data from laboratory rats [45], as well as observations on specimens in the reference collection, previously aged from cranial suture closure. The categories employed are early (distal humerus; distal tibiofibular), middle (distal ulna; proximal femur) and late (distal femur; proximal tibia, humerus and ulna) fusing elements.

As methods of murid species age assessment based on molar wear usually investigate the whole tooth row, adaptation of selected methods for the study of archaeological material was required. The scoring system for field mice *Apodemus sylvaticus* (based on the technique used for ageing yellow-necked mice *Apodemus flavicollis*; [46,47]) and house mice *Mus musculus* (based on [48,49]) was adjusted to make it suitable for single teeth and applicable to both murid species found in Orkney (table 2 for details). Scoring was done firstly for each recovered tooth row (complete or incomplete) as well as each separate tooth found. Once all the scores were known for the specific sample the MNI that could provide those scores was established, given visual similarity between the elements in terms of shape/size and same or very similar wear score. The most common tooth finds were the first molars, resulting in a relatively easy approximation of the final score for each individual. In more complex cases the approximation took third molar scores as more viable due to their being last to erupt, and scoring was adjusted accordingly.

**Table 2.** Summary of tooth wear levels used, with description and references for wear scoring used for field (as in yellow-necked mice [46]) and house mice [48]. Expected age relates to the information in the references and known information about the life cycle of both species (field mice in [50]; house mice in [51]). The system is most accurate when the third molar is present as it is the last to develop wear. Table from ([37] fig. 3.05).

wear level	description	…equals to:	expected age
0	no visible wear	1 and 2 in Lidicker [48]	< 1 month (juvenile)
		none or 1 in Adamczewska-Andrzejewska [46]	
1	wear areas on cusp tips, isolated from each other	3 in Lidicker [48]	1–2 months (sub-adult)
		1 or 2 in Adamczewska-Andrzejewska [46]	
2	wear areas on cusp tips, joining in between nearest cusps	4 in Lidicker [48]	3–4 months (adult)
		2 in Adamczewska-Andrzejewska [46]	
3	each row of cusps worn together	5 in Lidicker [48]	5–7 months (adult)
		3 in Adamczewska-Andrzejewska [46]	
4	singular wear surface, cusp morphology still identifiable	6 in Lidicker [48]	8–11 months (adult)
		4 in Adamczewska-Andrzejewska [46]	
5	singular wear surface, cusp morphology lost	7 and 8 in Lidicker [48]	12 + months (adult)
		5 in Adamczewska-Andrzejewska [46]	

Appendices with data or further examples, not included in the publication, are available as electronic supplementary material, data. For the site summary and key four contexts analysed and discussed in the paper, data display similar to Andrew's work [28], with detailed tooth loss, breakage and digestion counts, is available in the electronic supplementary material S1—Key Data. Data for each phase, context and sample recorded can be found in the electronic supplementary material S1—Phases/Contexts/Samples. Electronic supplementary material S1—References contains data adapted from Andrews ([28], pp. 209–213), a selection of predator assemblages used during the analysis as comparison with archaeological data. The selection included species currently or historically present on Orkney: barn owl *Tyto alba*, snowy owl *Bubo scandiacus* (formerly *Nyctea scandiaca*), long-eared owl *Asio otus*, short-eared owl *Asio flammeus*, kestrel *Falco tinnunculus*, peregrine *Falco peregrinus*, hen harrier *Circus cyaneus* and red fox *Vulpes vulpes*. Representative micrographs taken with the scanning electron microscope are shown in electronic supplementary material S2. All correlations and visualizations presented in the paper were coded in R (version 4.0.2) and run in RStudio (version 1.3.1073) [52,53]. All the code necessary to run the analysis and generate visualizations/tables is present in electronic supplementary material, S3.

## 3. Analysis

### 3.1. Taphonomy

In total, 4484 bone and tooth fragments belonging to at least 465 micromammals were retrieved from 205 contexts during the 1980s excavation seasons (table 3). From about 300 contexts that provided soil samples during both excavation seasons, 199 contained micromammal material. An additional six contexts were represented by hand-retrieved samples. The distribution of micromammal remains across the site and main time periods is not uniform. The majority of finds come from Phases 2 to 5, with waterlogged deposits reflecting early human activity within the area (Phase 2, AD eleventh century); the construction and utilization of two substantial buildings, firstly a ‘hall’ (Phase 3, AD twelfth century) and subsequently a ‘workshop’ (Phase 4, AD twelfth—fourteenth century); and then the collapse of buildings and gradual abandonment of the site (Phase 5, AD thirteenth—fifteenth century and later). It may reflect a genuinely higher density of skeletal fragments in these samples than in those coming from either natural soils pre-dating the Norse settlement (Phase 1, before AD eleventh century), or from layers that followed the settlement (agriculture in Phase 6, AD eighteenth–nineteenth century, kelp burning in Phase 7, AD nineteenth–twentieth century). Two hundred and eleven samples, reflecting 188 contexts, were retrieved from Phases 2 to 5, while Phase 1, 6 and 7 provided only evidence for 17 contexts. Blocks/contexts that provided the largest quantity of samples with micromammal remains were furnace and floor spreads within the workshop, walls and other contexts within the hall, and external deposits and abandonment layers (dumps) after abandonment of the workshop. Sampled windblown deposits in general rarely provided micromammal material, but given the methodology of 1980s excavations and restricted financing, a bias towards sampling of anthropogenic contexts cannot be excluded.

**Table 3.** Summary of NISP and MNI of all micromammal remains found at Tuquoy, considering major stratigraphy and individual species classes used during identification. More detail is provided in the electronic supplementary material S1.

phase	weight	no. samples	*Microtus*		*Apodemus*		*Mus*		unid. rodent		unid. mouse		*Sorex*		ΣNISP	ΣMNI
			NISP	MNI	NISP	MNI	NISP	MNI	NISP	MNI	NISP	MNI	NISP	MNI		
1	0.06	1	1	1	0	0	0	0	4	0	0	0	1	1	6	2
2	2.67	16	76	18	10	3	5	2	78	3	2	0	2	1	173	27
3	29.53	31	846	72	40	13	18	6	912	13	6	0	12	8	1834	112
4	33.43	130	546	119	63	29	76	35	1041	26	48	4	8	7	1782	220
5	7.44	34	119	28	25	16	35	19	372	14	17	0	2	2	570	79
6	1.06	7	14	5	0	0	0	0	32	1	1	1	1	1	48	8
7	1.12	9	22	8	0	0	5	2	39	6	4	0	1	1	71	17
Σ	75.31	228	1624	251	138	61	139	64	2478	63	78	5	27	21	4484	465

The evidence for possible disturbance was minimal. The only block with an identified intrusive rabbit burrow (Block 43) contained only one sample with a rodent bone (Context 1061). On the other hand, rabbit bones were indeed present in some sieved samples, but seem to be contemporary with other osseous remains found within these contexts.

Regardless of phase, most samples provided only 12 or fewer skeletal fragments, quite often only teeth, vertebrae or other small elements. For all samples, NISP median is just 8 and average abundances median is 5.59% when counting only skeletal elements and 6.37% when considering loose teeth, representing very incomplete specimens (table 4). Larger samples contained remains that were more skeletally complete, but most of them still contained a relatively small number of finds (table 4, Contexts). What may be interesting is that the most complete (average abundance for skeletal elements being 32%) sample came from Context 1112, a paving passageway infill from Phase 6, and belonged to a single Orkney vole. Similarly, Contexts 107, 200, 753, 1072 and 1078, found near the workshop/hall entrances or inside those buildings and belonging to Phase 4 or 5, provided samples with similar completeness.

**Table 4.** Summary of key data (NISP, MNI, species identified, average relative abundances and number of digested remains); for the site in general, samples (as mean/median values) and contexts discussed in the paper. More detail is provided in the electronic supplementary material, S1.

	data source			general counts			abundances, average		digestion, counts		
				ΣNISP	ΣMNI	species no.	only bones	incl. teeth	incisor	molar	hum & fem
general	site overall			4484	465	4	10.18%	11.98%	96	216	22
	samples	median		8	1.5	1	5.59%	6.37%	0	0	0
		mean		19.67	2.12	1.50	7.08%	8.22%	0.42	0.95	0.10
	context	phase	type								
key contexts	28	3	outdoor windblown sand with charcoal	83	5	3	20.71%	22.98%	8	10	0
	33	3	outdoor windblown sand with stones	1138	42	3	24.61%	30.44%	78	178	19
	1112	6	outdoor loam and stone infill	23	1	1	32.37%	29.54%	0	0	0
	1015	7	outdoor windblown sand	30	8	2	11.67%	10.42%	0	0	0
contexts mentioned	1022	1	outdoor sand layer	6	2	2	3.52%	4.58%	0	0	0
	279	2	outdoor layer with sand and charcoal	36	4	3	9.42%	12.48%	0	0	0
	306	2	outdoor soil infill	58	5	3	9.76%	13.32%	0	0	0
	1171	2	outdoor sand infill	6	2	2	3.33%	3.68%	0	0	0
	99	3	stone wall	22	3	3	7.03%	8.66%	0	1	0
	226	3	indoor floor deposit	43	3	2	10.90%	14.03%	0	0	0
	1205	3	outdoor windblown sand with charcoal	35	3	1	8.24%	8.58%	0	1	0
	96	4	outdoor soil deposit	79	4	3	16.07%	22.88%	1	1	1
	107	4	entrance soil and rubble deposit	84	7	2	15.13%	16.99%	0	0	0
	context	phase	type								
	200	4	entrance stone passageway	47	5	2	15.03%	15.62%	0	0	0
	737	4	indoor hearth	2	1	1	3.33%	4.41%	1	0	0
	753	4	indoor loam infill	51	5	2	20.84%	18.68%	0	0	0
	1020	4	abandonment soil and stone deposit	14	2	1	5.09%	7.43%	0	0	0
	1072	4	indoor loam infill	45	3	2	24.75%	22.00%	0	0	0
	108	5	later structure, windblown sand with stones	24	3	3	11.62%	12.70%	0	0	0
	137	5	abandonment rubble	18	4	4	5.11%	6.35%	0	0	0
	1078	5	abandonment rubble	27	3	3	30.00%	26.47%	0	0	0
	1101	7	outdoor windblown sand	1	1	1	3.33%	2.94%	0	0	0

Among the largest contexts in terms of NISP were Context 33 and 28, both from Block 29, Phase 3. Context 33 provided 1138 NISP, 42 individuals in total, about a quarter of the total number of micromammal finds at Tuquoy and 9% of all MNI. In turn, Context 28 contained 83 NISP, representing five individuals. Contextually, both were closely associated with each other, creating a larger assemblage of micromammal remains. While sporadic staining of bone or tooth surfaces was noted in some samples, especially on those retrieved from material-rich sediments, bones from Block 29 were evenly stained in dull to dark brown colours, denoting a shared taphonomic history. It is notable that both contexts provided average abundances above 20%, much higher than the Tuquoy median or even other large contexts and similar to values known from Skara Brae accumulations [9,14].

Skeletal elements and loose teeth NISP values, relative abundances and fragmentation patterns of the selected samples (Contexts 28, 33, 1015 and 1112) were compared with known neotaphonomic assemblages created by predators (table 5). For the first two data groups (elements NISPs and relative abundances), both Contexts 28 and 33 provided similar correlation coefficients with references, most notably significant (considering d.f. = 15) with kestrels and hen harriers, and slightly less significant with red foxes. Weak but relatively high correlations were also noted for peregrines. Context 1015 NISP did not match any known assemblage type, with relative abundances showing a significant correlation with six out of eight species, the highest being with short-eared owls. It was perhaps due to very low NISP values affecting the results, as Andrews' [28] assemblages were much larger (smallest assemblage NISP equalling 170, while 1015 being only 30). Relative abundances were also generally lower, but due to being on a theoretically finite scale (0 to 1, or 0% to 100%), the effect was not as visible, thus resulting in significant results. The low NISP count most likely also affected correlations in the case of Context 1112, with additional bias coming from high relative abundances due to MNI used for calculating them being only one. For NISP, only two correlations with owl species could be matched as significant. However, for relative abundances, five out of eight correlations came out as significant, the highest being with hen harriers. However, such large differences between correlations make the results dubious in the case of both contexts. Long bone breakage shows some tendency towards diurnal raptors and foxes, with values for Contexts 33 and 1015 exhibiting strong correlation. However, correlations for other contexts are very low, often negative, again probably showing the effect of a small sample pool.

**Table 5.** Comparison between samples from the discussed contexts and examples of assemblages made by specific predators (selection taken from [28, pp. 209–213], selected data enclosed in electronic supplementary material, S1, References; no comparable bone breakage data for peregrines).

context	reference data from Andrews [28]							
	barn owl	snowy owl	long-eared owl	short-eared owl	kestrel	peregrine	hen harrier	red fox
Corr. Pearson, NISP, d.f. = 15								
Context 33	*r* = 0.187 *p* = 0.473	*r* = −0.077 *p* = 0.769	*r* = 0.142 *p* = 0.586	*r* = 0.175 *p* = 0.502	*r* = 0.685 *p* = 0.002	*r* = 0.395 *p* = 0.117	*r* = 0.547 *p* = 0.023	*r* = 0.274 *p* = 0.287
Context 28	*r* = 0.168 *p* = 0.519	*r* = −0.119 *p* = 0.649	r = 0.154 *p* = 0.554	*r* = 0.142 *p* = 0.586	*r* = 0.619 *p* = 0.008	*r* = 0.325 *p* = 0.204	*r* = 0.511 *p* = 0.036	*r* = 0.215 *p* = 0.407
Context 1015	*r* = −0.269 *p* = 0.296	*r* = −0.295 *p* = 0.251	*r* = −0.202 *p* = 0.437	*r* = −0.197 *p* = 0.448	*r* = −0.079 *p* = 0.762	*r* = −0.157 *p* = 0.549	*r* = −0.028 *p* = 0.916	*r* = −0.221 *p* = 0.393
Context 1112	*r* = 0.640 *p* = 0.006	*r* = 0.221 *p* = 0.395	*r* = 0.683 *p* = 0.003	*r* = 0.460 *p* = 0.063	*r* = 0.252 *p* = 0.329	*r* = 0.322 *p* = 0.207	*r* = 0.039 *p* = 0.881	*r* = 0.303 *p* = 0.237
Corr. Pearson, relative abundances, d.f. = 15								
Context 33	*r* = 0.050 *p* = 0.849	*r* = −0.358 *p* = 0.158	*r* = 0.241 *p* = 0.352	*r* = 0.313 *p* = 0.221	*r* = 0.695 *p* = 0.002	*r* = 0.479 *p* = 0.052	*r* = 0.786 *p* = <0.001	*r* = 0.529 *p* = 0.029
Context 28	*r* = 0.322 *p* = 0.207	*r* = 0.033 *p* = 0.901	*r* = 0.517 *p* = 0.033	*r* = 0.525 *p* = 0.031	*r* = 0.737 *p* = 0.001	*r* = 0.430 *p* = 0.085	*r* = 0.716 *p* = 0.001	*r* = 0.574 *p* = 0.016
Context 1015	*r* = 0.572 *p* = 0.017	*r* = 0.392 *p* = 0.119	*r* = 0.595 *p* = 0.012	*r* = 0.754 *p* = <0.001	*r* = 0.597 *p* = 0.011	*r* = 0.641 *p* = 0.006	*r* = 0.344 *p* = 0.176	*r* = 0.584 *p* = 0.014
Context 1112	*r* = 0.507 *p* = 0.038	*r* = 0.167 *p* = 0.521	*r* = 0.575 *p* = 0.016	*r* = 0.571 *p* = 0.017	*r* = 0.634 *p* = 0.006	*r* = 0.302 *p* = 0.239	*r* = 0.665 *p* = 0.004	*r* = 0.088 *p* = 0.738
Corr. Pearson, long bone breakage, d.f. = 13								
Context 33	*r* = −0.527 *p* = 0.044	*r* = −0.439 *p* = 0.101	*r* = −0.514 *p* = 0.050	*r* = −0.497 *p* = 0.059	*r* = 0.597 *p* = 0.019		*r* = 0.293 *p* = 0.290	*r* = 0.824 *p* = <0.001
Context 28	*r* = −0.172 *p* = 0.540	*r* = −0.157 *p* = 0.575	*r* = −0.178 *p* = 0.525	*r* = −0.158 *p* = 0.574	*r* = 0.292 *p* = 0.292		*r* = 0.318 *p* = 0.248	*r* = 0.340 *p* = 0.216
Context 1015	*r* = 0.020 *p* = 0.944	*r* = 0.018 *p* = 0.948	*r* = 0.026 *p* = 0.926	*r* = 0.057 *p* = 0.841	*r* = 0.564 *p* = 0.028		*r* = 0.585 *p* = 0.022	*r* = 0.376 *p* = 0.167
Context 1112	*r* = −0.005 *p* = 0.987	*r* = 0.071 *p* = 0.802	*r* = −0.008 *p* = 0.979	*r* = 0.004 *p* = 0.988	*r* = 0.402 *p* = 0.138		*r* = 0.063 *p* = 0.823	*r* = 0.149 *p* = 0.597

**Figure 2.**
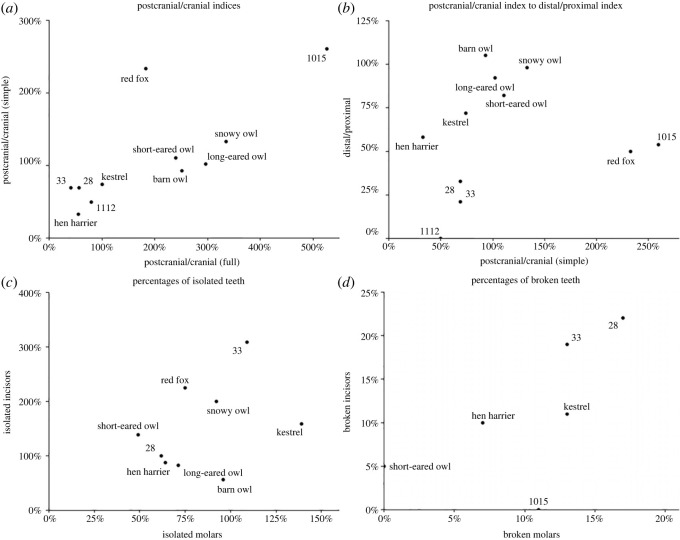
Comparisons between three indices regarding proportions between different anatomical regions (*a*) and (*b*), and ratios of isolated (*c*) and broken (*d*) teeth. See table 1 for indices explanation. Selection of species taken from [28], pp. 209–213, selected data enclosed in the electronic supplementary material, S1, References.

For various indices proposed by Andrews [28], the four contexts partially relate to species used for the comparison (figure 2). The proportions between postcranial and cranial elements clearly show the similarity of Contexts 28, 33 and 1112 to kestrel and hen harrier assemblages. Context 1015 strongly differs from all the indices, with the nearest association being red fox or, more distantly, the snowy owl. When considering proportions between distal to proximal limb elements, however, the predominance of proximal long bones (i.e. humeri and femora) in the archaeological contexts can be seen. This trend makes Context 1015 most similar to red foxes, with other contexts being further away (Context 28 and 33) or even lacking distal elements to compare (Context 1112). Only Context 28 and 33 could be compared when considering teeth. While Context 28 shows percentages of isolated teeth more or less within the scope of species comparisons, and very similar to hen harrier, Context 33 provides a much higher percentage of isolated incisors than species comparisons. When considering tooth breakage, both contexts incisor breakage is higher than species comparisons, with 28 higher also in terms of molar breakage. Molar breakage in the case of Context 33 shows similarity to that noted for kestrels.

More conclusive data derive from assessment of digestion marks on rodent incisors, teeth and epiphyses of proximal long bones, generally for all the contexts and in detail for key assemblages. The overwhelming majority of skeletal elements altered by digestion were from Contexts 33 and 28 (table 4, key contexts), including all of those molars considered as heavily digested. Percentages of digested incisors and molars as well as scores ranging from light to extreme digestion suggest a category 4 predator (little owl, kestrel and peregrine) and, to a lesser degree, one from category 3 (European eagle owl, tawny owl and little owl) (see table 3.14 and related chapter in [28]). Additionally, 25 samples with single teeth or long bones altered by digestion have also been found, mostly from outdoor contexts of Phase 3 and early floor deposits of Phase 4. However, other types of taphonomic alterations, notably weathering and abrasion, were also prevalent in the samples. See electronic supplementary material, S2 for selected micrographs.

### 3.2. Taxonomic composition

Orkney voles form over half of the micromammal population observed (MNI 251), followed by field and house mice (MNI 61 and 64; table 3). Pygmy shrews *Sorex minutus* appeared in 19 samples (MNI 21). About five MNI were attributed to mice but not to specific species, while 63 could only be described as unidentified rodent. Only one context (137) contains all of the species present, an early rubble deposit from Phase 5, with MNI of one for each of the four species. Those that contain three species differ in species composition between Phase 3 and later phases. Both Contexts 28 and 33 (Phase 3), contained voles, wood mice and pygmy shrews in windblown sand co-mingled with products of human activity like rubble or charcoal. All but one of the later contexts include a mixture of voles and two murid species, with the contexts themselves being of anthropogenic or mixed origin. Other faunal remains and some carbonized material, in two cases cereal grain, were found within them.

**Figure 3.**
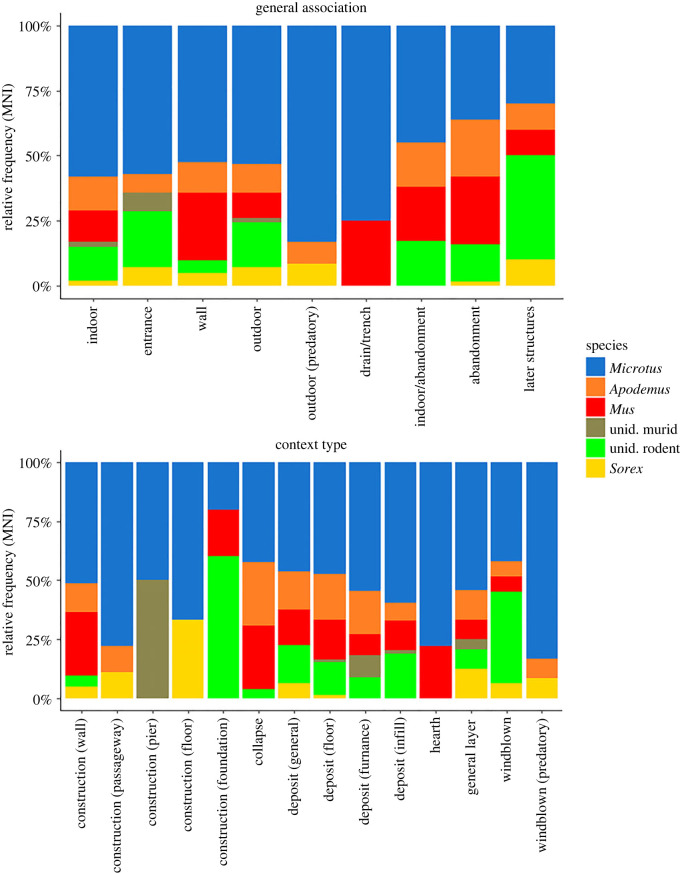
MNI values for specific species classes in relation to general location of the context (upper plot) and primary type of context (lower plot).

All of the species identified still inhabit Westray today and are considered a stable element of the island microfauna; no temporary introductions from modern times (e.g. black rats) were identified [1,8,54]. Some differences between species could be seen both temporally and spatially (for the latter figure 3). Orkney voles were present from Phase 1 (Context 1022) through to Phase 7 (contexts from Episode 26) and comprise the majority of finds in each of the seven phases. Their bones were found both inside the studied structures, within specific constructions and in a variety of outside contexts, in all context types. Voles were especially commonplace in larger assemblages in terms of both MNI and percentage of the Context MNI attributed to those species. In the case of Context 33, 36 (86%) out of 42 identified micromammals were voles. Some of the larger vole assemblages, including some containing digested molars, came from floor spreads of the workshop.

By contrast, field mice first appear in Phase 2, Context 1171, an early pit infill, and are present on the site until Phase 5 (Context 108). Most finds are heavily scattered, representing at best one or two individuals when counted on the context level. The biggest concentration found contained only three, in Context 33. On the block level, Phase 4 floor spreads within the workshop provided higher numbers of field mice. Contexts on the outside and within the hall entrance, as well as occasionally walls themselves, provided scattered remains of the field mice, pointing towards their likely presence across the wider area. Abandonment layers from Phase 5 also confirm field mice to be inhabiting the site during and shortly after its abandonment, but later Phases (6 and 7) did not provide any evidence of them.

House mice appeared slightly later than field mice, in Context 306 (Block 13, Phase 2), a pit containing organic material and charcoal. Similarly to field mice, their remains usually represent single individuals, with some relationship with floor layers. Noticeable exceptions are Blocks 77 and 97, with a relatively high number of house mice in either outside contexts or midden/rubble deposits, and a single bone retrieved from a drain/trench context. There are no remains from Phase 6, apart from a few murid bones unidentified to taxon, but house mice can once again be seen in Block 106 (Phase 7), within the windblown sands.

Finally, pygmy shrew bones appeared in Block 2, Context 1022, in natural sediments, and appear sporadically until Context 1101, Phase 7. Most finds from Phase 3 were single finds retrieved from the construction contexts, but natural sands and previously identified accumulations (Contexts 28 and 33) also provided a number of different fragments.

### 3.3. Age and size

Long bones show all stages of growth but in general there is a tendency towards sub-adults with early and middle fusing epiphyses which are already fused (table 6). Based on skeletal growth, older pygmy shrews and murids were present, while a greater proportion of the voles were sub-adult specimens. The unfused humeri and tibiae of micromammal species are potentially important, because the presence of juvenile specimens may be a sign of nesting and/or burrowing within the site although not necessarily within the excavated area. The earliest find of a humeral shaft with no evidence for epiphyseal fusion came from a sandy layer, Context 279 (Phase 2), pre-dating the hall. It was also the only case of the early fusing epiphyses identified to a species, specifically a vole. In Phase 3, unfused cases of the early and middle stages appeared in Contexts 226 (a primary floor layer within the hall) and 1205, both mixtures of sandy and clay soils with remains of human activity. More unfused finds appear in Phase 4 but mostly outside of the hall and workshop, for example Contexts 96 and 107, both from the same Block 77. Unfused epiphyses attributable to the middle and late stage, and to voles and mice species, appear in relatively high numbers in Phase 5, specifically Block 97, but none was retrieved from Phase 6. The last finds are proximal unfused femora within Phase 7, Block 106.

**Table 6.** Summary of long bone fusion for each species class recorded in Tuquoy.

epiphysis		overall		unidentified		*Microtus*		*Apodemus*/*Mus*		*Sorex*	
		unfused	fused	unfused	fused	unfused	fused	unfused	fused	unfused	fused
early											
humerus—distal		14	129	13	65	1	42	0	19	0	3
tibia—distal		3	56	3	33	0	12	0	8	0	3
middle											
ulna—distal		5	17	5	7	0	6	0	4	0	0
femur—proximal		23	134	14	72	4	52	5	8	0	2
late											
ulna—proximal		42	23	42	0	0	17	0	6	0	0
humerus—proximal		50	16	20	7	26	7	2	2	2	0
femur—distal		36	17	19	9	13	2	4	3	0	3
tibia—proximal		41	10	18	2	15	3	7	3	1	2

**Figure 4.**
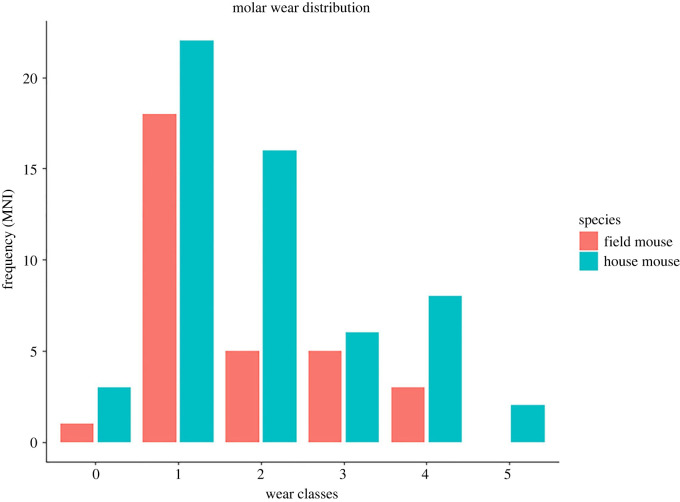
Summary of molar tooth wear for murids found at Tuquoy, as frequency of species MNI.

Dental wear reveals the presence of murids of various ages, from the unworn molars of juvenile animals to the extreme attrition found in old individuals (figure 4). Results of the scoring enabled assessment of 57 individuals for house mice, about 84% of the estimated MNI. By contrast, 32 individuals could be scored for field mice, about 50% of the MNI estimation, probably due to a lesser proportion of teeth being retrieved than in the case of house mice, in conjunction with a larger proportion of bones attributable to the species. Nevertheless, tooth wear data supplements the long bone fusion data, showing that young house mice are present in Phases 3 to 5. In the case of both mandibular and maxillary molar wear, most finds lie within the scores of 1 to 4, reflecting specimens up to 11 months old. House mice provided sufficient numbers of unworn teeth to be sure of the presence of at least three juvenile individuals and enough with heavy wear to indicate the presence of at least two overwintered animals in the sample. By contrast, field mice mostly belonged to a single age category (1) with some older specimens occasionally present. Only one possible juvenile was found.

## 4. Discussion

### 4.1. Deposition

There is strong evidence for predatory activity in Phase 3. Archaeologically, predatory activity is usually visible as sets of remains reflecting, in terms of composition, preservation and location, a specific predatory behaviour [27,28]. In Orkney, the predators are almost exclusively avian, from a number of nocturnal owl species to diurnal raptors, such as kestrels, peregrines or hen harriers [1,54]. Almost all those species are known to regurgitate pellets containing partially or undigested remains. Especially in the case of roosting or nesting sites, the place of deposition may contain hundreds of individual pellets, densely packed together. In the case of the Tuquoy samples, which usually include only about 10 l from each context, one sample taken from Context 33 definitely represents a dense group of micromammal remains, with two additional bones of a small passerine bird and one of a gull (S Hamilton-Dyer October 2017–June 2018, personal communication).

The quantified data and taphonomic marks on bones (electronic supplementary material, S1, Key Data), especially those attributable to digestion, probably exclude most of the nocturnal owls as well as terrestrial carnivores, despite the presence of both owl remains (S Hamilton-Dyer October 2017–June 2018, personal communication) and domestic cat (*Felis catus*) bones within Tuquoy strata. Rather, it points towards diurnal raptors [55], especially kestrels. These birds are known to roost and nest on rock faces along the coast, sometimes on human structures [56]. The location of Contexts 28 and 33, in the flagged passageway, may represent such a situation. The Context 33 sample contained mainly voles together with some field mice and birds, similar to kestrel assemblages studied by Reynolds [57].

However, kestrel remains have not been found at Tuquoy. Among diurnal raptors, only peregrine bones were found, within Phase 4 (Context 1020) (S Hamilton-Dyer October 2017–June 2018, personal communication), but peregrines predominantly deposit bird bones and are rarely responsible for substantial micromammal assemblages ([28], p. 196). Correlations discussed in the analysis also exclude them as potential depositors. While hen harriers are correlated with major assemblages almost as well as kestrels, there should be a more marked digestion pattern present at the site. Other raptors found in the assemblage were buzzards *Buteo buteo* and white-tailed eagles *Haliaeetus albicilla*, but both of these species would produce even more heavily digested remains, similar to those from the hen harrier, and these would also contain a very different set of taxa.

There are some methodological issues that could have biased the results, making comparisons with Andrews' [28] data less reliable. Some species may show seasonal variation in some taphonomic changes, most notably differences in digestion scoring between winter and other seasons, which are known from owl assemblages [58]. Another possible issue impacting digestion scoring may be due to the different structure and shape of skeletal elements as well as teeth between different kinds of micromammals, especially between arvicolids, murids and soricids. The methodology was originally established on accumulations containing predominantly vole species [28], with detailed investigation into non-arvicolid digestion considered more recently [36]. The first issue is hard to assess for diurnal raptors, with more severe seasonal digestion coming from owls still likely to be excluded. The second issue should not be a problem for Contexts 28 and 33, mainly because of the predominance of voles and only a minor presence of field mice. Evidence pointing towards additional taphonomic history after deposition, such as heavy abrasion and weathering, however, may bias the assessment. It is therefore not possible to identify the predator involved here beyond labelling it as a ‘diurnal raptor’ and category 4 predator according to the aforementioned Andrews [28] scale.

Considering that there is evidence of digestion in a number of samples that are much smaller than those from Context 33 or 28, along with specific brownish staining, it is possible that more predator assemblages were present on the site. Those samples may represent scattering of remains from these assemblages. Archaeological evidence for the dispersal of micromammal remains from the primary predatory assemblage is known (e.g. [59]) and bone dispersal generally forms an important part of taphonomic studies ([60], pp. 114–222). Disturbance of primary assemblages was most likely due to human agency, probably connected with everyday activity, as well as hall and workshop construction and maintenance. Single-digested teeth are present within building features, such as the hall wall (Context 99, Phase 3), hearth ash (Context 737, Block 46, Phase 4) and workshop spreads during its early utilization (Block 47, Phase 4). Considering the lack of primary assemblages after Phase 3 and that almost all dispersed samples come from Phases 3 and 4, predator activity on the fringe of the settlement may have moved in response to the construction of the workshop. However, it is possible that limited sampling during the first excavation season simply omitted other predatory activity within the site or downplayed the importance of some contexts in this regard. It is also possible that some digestion could not be noted or was underscored, as especially light and moderate digestion on murid molars can be difficult to identify while soricid teeth show no equivalent to light digestion [36].

Aside from predation, the presence of undigested and unstained remains in the majority of samples may reflect self-trapping of micromammals or natural death and subsequent dispersal of their remains over a wider area. Self-trapping in man-made features is known to occur in archaeological sites, sometimes concentrating high numbers of individuals in a relatively short time (see [27,61]). A paved passageway could be a barrier for micromammals, thus creating samples such as that from Context 1112 (Block 99, Phase 6). Such finds, if not disrupted after deposition, are usually relatively complete. Similar samples to Context 1112 were also found near the entrances to the hall and workshop. Considering the presence of old individuals (those with fully fused late-fusing bones, dental attrition around 4 and 5), one may also infer the natural death of individual micromammals, most likely during winter when a lack of food puts a huge stress on these populations.

### 4.2. Taxonomic composition and population dynamics

All four taxa encountered at Tuquoy exhibit different kinds of relationships with humans and the anthropic environment [29]. Orkney voles may represent an example of environmental synanthropism, the ability to take advantage of a human-modified ecosystem, such as agricultural fields or pastures. As mentioned previously, this species may have been brought to Orkney intentionally. They currently inhabit mostly rough grassland, with smaller populations in other habitats [1,57]. However, such species can also use non-intensified crop or pasture land and are known to have done so in Orkney until the early AD twentieth century [62]. During the last 200 years, voles have not been considered as serious pests by local farmers and no intentional means of pest control have been applied for regulating their populations (see [57,62]). At Tuquoy, given the presence of juvenile bones but the absence of elaborate vole burrows in the stratigraphy, it is possible that voles were active on the site fringes, occasionally ‘foraging’ deeper into the site. However, a significant proportion of the finds came from predator assemblages, or was once a part of one, and the presence of predators themselves usually discourages scavengers from both roaming or establishing colonies.

Pygmy shrews often show edificarian or domiciliary synanthropism (living within human-made structures), with some evidence pointing towards it in the case of Tuquoy. These animals are solitary species, with a high tolerance towards suboptimal environments but rarely appearing in densities higher than two or three individuals per hectare [63]; in the British Isles, they are occasionally found within houses, living on upper floors or attics and feeding on insects there, and in old stone walls [64]. Studies indicate that their introduction to Orkney most likely happened in the Late Neolithic, firstly to Orkney mainland and then to Westray [26]. However, the Tuquoy site provides the earliest physical evidence of their presence in Orkney (Phase 1, earlier than AD 1000). Known samples from Birsay Bay seem to be of similar or slightly younger age ([65], pp. 64–67, 96–100, 147–156, 161–191). It may be that their introduction indeed occurred somewhere between the Early Bronze to Early Norse Period, and by the AD tenth–eleventh century, the population had already settled a number of Orcadian islands. Samples from Contexts 99 and 176, parts of the original hall walls (Phase 3, AD twelfth century), and Context 244, an outside context contemporary to the hall, suggest some sort of correlation with human activity. Moreover, shrew bones from those samples were structurally intact and did not show any visible taphonomic marks, such as digestion or weathering, on their surface. However, similar quantities of remains as in the indoor and construction contexts were also retrieved from outdoor contexts, including those of a possible predatory origin, perhaps pointing towards their presence also in the wild.

Field mice are a good example of a wild micromammal that under various conditions can exhibit all known synanthropic relationships, including seasonal or full commensalism, feeding on human-stored organics, especially food. This species can live in a variety of woodland and grassland habitats as well as pastures and agricultural fields [50]; field mice are also capable of infesting human habitation, especially if other competitors are absent, but this mainly occurs during winter, when the mice are driven to search for new food sources. In these circumstances, field and house mice may coexist alongside each other, in and around human dwellings. Field mice were introduced to Orkney long before the settlement at Tuquoy was established, given their presence in Neolithic contexts from Skara Brae [3,6,9]. However, it is not known whether they were introduced to Westray at the same time as to other parts of the archipelago, although data from Sanday and Mainland indicate similar dates of introduction. A mandible and maxilla belonging to this species were identified in Links of Noltland sieves but whether those were intrusive or contemporary finds to other Neolithic finds is yet to be confirmed. The presence of field mice in predator assemblages from Tuquoy, along with digestion and discoloration, indicates that their population was already established in wild or semi-wild habitats. Other finds provide information about their activity on the site, including its abandonment in Phase 5, but given the age data, with just one juvenile and no overwintered specimens, may point towards seasonality rather than a constant presence. Moreover, field mice seem to be completely absent in the last two phases and, while it may be due to a sampling bias, it may also be due to changes in land management (such as agriculture and kelp burning) or other environmental factors.

In the case of Tuquoy, house mice seem to be a fully commensal species. While house mice can sometimes thrive in wild environments, they tend to occupy fully commensal niches in locations outside their original range [51], as their evolutionary path and recent expansion throughout the world were clearly enabled by their adaptation to human presence [66,67]. While there is little information available regarding historical populations of house mice in Orkney, research on archaeological material from Shetland revealed their presence in middle Iron Age deposits (second century BC–AD fourth century [68]). However, this does not rule out later introductions, including Viking involvement in their movement across the North Sea and beyond. The lack of identifiable house mouse remains in predator assemblages from Tuquoy correlates with Reynolds' observations [57] and presumably reflects their close association with human habitations and the low numbers that will be available to predators hunting in the natural environments that surround them. The first appearance of house mice here, correlating with early human activity, and the presence of juvenile as well as overwintered individuals within what are predominantly anthropomorphic contexts, reflect their commensal lifestyle and are consistent with the foregoing interpretation. In particular, the many juvenile remains recovered from the midden deposits in Phase 5 strongly suggest that house mice were nesting within the human settlement. The latest appearance of house mice, in windblown sand from Phase 7, is more likely due to scattering of their remains from older deposits, or association with some form of human activity being carried out in the vicinity of the site, rather than the adaptation of mice to a more natural environment.

## 5. Conclusion

The Tuquoy excavation has provided sufficient material to draw several conclusions. Taphonomic alterations suggest predatory activity within the early settlement site, possibly coming from a medium-sized diurnal raptor, but other factors most likely played an equal part in micromammal deposition, in particular accidental self-trapping within man-made features or even natural death of species living in the vicinity of the site. Still, evidence for additional non-predatory taphonomic changes suggests more work is needed to be certain of predatory deposition and proper species associations. The taxonomic composition reflects the impact of humans on the microfaunal diversity of remote insular environments, which are otherwise generally isolated from the natural dispersal and migrations of animals within continental landmasses. The four species identified among the remains reflect different responses to the human-made environment, from dwelling within the agrarian landscape to seasonal or complete commensalism. The research uncovers especially interesting information about the previously neglected pygmy shrews and field and house mice, especially the evidence for their introduction to Westray.

However, more research has to be done on archaeological samples from Orkney and other parts of Britain to properly understand the population history and dynamics of micromammal species, especially in relation to human activity. The human impact on the insular environment is especially visible in the case of Orkney voles, showing how their introduction by humans during the Neolithic might have permitted the establishment of new predator–prey relationships. This event possibly prompted some species to colonize Orkney in search of such prey, as a recent decline in the vole population [54] is strongly reflected in a decline of all species hunting them [1,54]. Isotope analysis may show changes within the subsistence and economy of both humans and micromammals [69,70] and may possibly differentiate between commensal and non-commensal species.

## Data Availability

Available online data supporting this paper include an Excel data file with appendix data (electronic supplementary material, S1), a PDF containing selected SEM micrographs alongside description (electronic supplementary material, S2) and an R script project (zipped) with short statistical and visual analysis (electronic supplementary material, S3) [71].

## References

[1] Berry RJ. 2000 Orkney nature. London, UK: Academic Press.

[2] Corbet GB. 1961 Origin of the British insular races of small mammals and of the ‘Lusitanian’ Fauna. Nature **191**, 1037-1040.

[3] Corbet GB. 1979 Appendix E: the rodent remains. In Investigations in Orkney (ed. C Renfrew), pp. 135-137. London, UK: Society of Antiquaries of London.

[4] Corbet GB. 1986 Temporal and spatial variation of dental pattern in the voles, *Microtus arvalis*, of the Orkney Islands. J. Zool. **208**, 395-402.

[5] Haynes S, Jaarola M, Searle JB. 2003 Phylogeography of the common vole (*Microtus arvalis*) with particular emphasis on the colonisation of the Orkney archipelago. Mol. Ecol. **12**, 951-956. 12753214 10.1046/j.1365-294x.2003.01795.x

[6] Nicholson RA. 2007 Small mammals. In Investigations in Sanday, Orkney Vol. 2: Tofts Ness, Sanday. An island landscape through three thousand years of prehistory (ed. SJ Dockrill), pp. 206-208. Kirkwall, UK: The Orcadian Press & Historic Scotland.

[7] Martínková N et al. 2013 Divergent evolutionary processes associated with colonization of offshore islands. Mol. Ecol. **22**, 5205-5220. 23998800 10.1111/mec.12462 PMC4159590

[8] Herman JS, Jóhannesdóttir F, Jones EP, McDevitt AD, Michaux JR, White TA, Wójcik, JM, Searle JB. 2016 Post-glacial colonization of Europe by the wood mouse, *Apodemus sylvaticus*: evidence of a northern refugium and dispersal with humans. Biol. J. Linnean Soc. **120**, 313-332. (10.1111/bij.12882)

[9] Romaniuk AA, Shepherd AN, Clarke DV, Sheridan AJ, Fraser S, Bartosiewicz L, Herman JS. 2016 Rodents: food or pests in Neolithic Orkney*.* R. Soc. Open Sci. **3**, 160514. (10.1098/rsos.160514) 27853568 PMC5098993

[10] Gorman ML, Reynolds R. 2008 Orkney and Guernsey vole. In Mammals of the British isles: handbook (eds S Harris, DW Yalden), pp. 107-110, 4th edn. Southampton, UK: The Mammal Society.

[11] Cucchi T et al. 2014 The changing pace of insular life: 5000 years of microevolution in the Orkney vole (*Microtus arvalis orcadensis*). Evol. Int. J. Org. Evol. **68**, 2804-2820. 10.1111/evo.12476 PMC5366975 24957579

[12] Thaw (née Haynes) S, Jaarola M, Seatle JB, Dobney KM. 2004 The origin of the Orkney vole *Microtus arvalis orcadensis*: a proxy for reconstructing human movements. In Atlantic connections and adaptations: economies, environments and subsistence in the North Atlantic (eds R Housley, G Coles), pp. 114-119. Oxford, UK: Oxbow Books.

[13] Sheridan JA, Pétrequin P. 2014 Constructing a narrative for a Neolithic of Britain and Ireland: the use of ‘hard science’ and archaeological reasoning. In Early farmers: the view from archaeology and science; Proc. of the British Academy 198. (eds A Whittle, P Bickle), pp. 369-390. Oxford, UK: Oxford University Press.

[14] Romaniuk AA, Shepherd AN, Clarke DV, Sheridan AJ, Fraser S, Bartosiewicz L, Herman JS. 2016 Data from: Rodents: food or pests in Neolithic Orkney. Dryad Digital Repository. (10.5061/dryad.j333r) PMC5098993 27853568

[15] Romaniuk AA et al. 2020 Combined visual and biochemical analyses confirm depositor and diet for Neolithic coprolites from Skara Brae. Archaeol. Anthropol. Sci. **12**, 274. (10.1007/s12520-020-01225-9)

[16] Brothwell DR. 1981 The Pleistocene and Holocene archaeology of the house mouse and related species. Symp. Zool. Soc. Lond. **47**, 1-13.

[17] O'Connor TP. 2010 The house mouse. In Extinctions and invasions: a social history of British fauna (eds T O'Connor, NJ Sykes), pp. 127-133. Oxford, UK: Oxbow Books.

[18] Cucchi T et al. 2013 On the trail of Neolithic mice and men towards Transcaucasia: zooarchaeological clues from Nakhchivan (Azerbaijan). Biol. J. Linn. Soc. **108**, 917-928. (10.1111/bij.12004)

[19] Weissbrod L, Marshall FB, Valla FR, Khalaily H, Bar-Oz G, Auffray JC, Vigne JD, Cucchi T. 2017 Origins of house mice in ecological niches created by settled hunter-gatherers in the Levant 15,000 y ago. Proc. Natl Acad. Sci. USA **114**, 4099-4104. (10.1073/pnas.1619137114) 28348225 PMC5402403

[20] Cucchi T et al. 2020 Tracking the Near Eastern origins and European dispersal of the western house mouse. Sci. Rep. **10**, 8276. (10.1038/s41598-020-64939-9) 32427845 PMC7237409

[21] Armitage PL. 1994 Unwelcome companions: ancient rats reviewed. Antiquity **68**, 231-240.

[22] Reilly K. 2010 The black rat. In Extinctions and invasions: a social history of British fauna (eds T O'Connor, NJ Sykes), pp. 134-145. Oxford, UK: Oxbow Books.

[23] Yu, H et al. 2022 Palaeogenomic analysis of black rat (*Rattus rattus*) reveals multiple European introductions associated with human economic history. Nat. Commun. **13**, 2399. (10.1038/s41467-022-30009-z) 35504912 PMC9064997

[24] Ledevin, R et al. 2016 Phylogeny and adaptation shape the teeth of insular mice. Proc. R. Soc. B **283**, 20152820. (10.1098/rspb.2015.2820) PMC4760175 26842576

[25] Chevret P, Hautier L, Ganem G, Herman J, Agret S, Auffray JC, Renaud S. 2020 Genetic structure in Orkney island mice: isolation promotes morphological diversification. Heredity **126**, 266-278. (10.1038/s41437-020-00368-8) 32980864 PMC8027461

[26] Vega Bernal R. 2010 Phylogeographic and morphometric studies on Eurasian pygmy shrew sorex minutus: insights into its evolutionary history and postglacial colonisation in Europe. PhD Thesis, The University of York, UK. EthosID: uk.bl.ethos.533462.

[27] Stahl PW. 1996 The recovery and interpretation of microvertebrate bone assemblages from archaeological contexts. J. Archaeol. Method Theory **3**, 31-75.

[28] Andrews P. 1990 Owls, caves and fossils. Chicago IL: University of Chicago Press.

[29] O'Connor T. 2013 Animals as neighbours: the past and present of commensal species. East Lansing MI: Michigan State University Press.

[30] Hulme-Beaman A, Dobney K, Cucchi T, Searle JB. 2016 An ecological and evolutionary framework for commensalism in anthropogenic environments. Trends Ecol. Evol. **31**, 633-645. 27297117 10.1016/j.tree.2016.05.001

[31] Avery DM. 1982 Micromammals as paleoenvironmental indicators and an interpretation of the Late Quaternary in the Southern Cape Province. Ann. South Afr. Mus. **85**, 183-374.

[32] Long JR. 2003 Introduced mammals of the world: their history, distribution and influence. Wallingford, UK: CABI Publishing.

[33] Cuthbert R, Hilton G. 2004 Introduced house mice *Mus musculus*: a significant predator of threatened and endemic birds on Gough Island, South Atlantic Ocean? Biol. Conserv. **117**, 483-489.

[34] Fernández-Jalvo Y, Andrews P, Sevilla P, Requejo V. 2014 Digestion versus abrasion features in rodent bones. Lethaia **47**, 323-336. (10.1111/let.12061)

[35] Fernández-Jalvo Y, Andrews P. 2016 Atlas of taphonomic identifications: 1001+ images of fossil and recent mammal bone modification. New York, NY: Springer.

[36] Fernández-Jalvo Y, Andrews P, Denys C, Sesé C, Stoetzel E, Marin-Monfort D, Pesquero D. 2016 Taphonomy for taxonomists: implications of predation in small mammal studies. Quat. Sci. Rev. **139**, 138-157.

[37] Romaniuk AA. 2022 Rethinking established methodology in micromammal taphonomy: archaeological case studies from Orkney, UK (4th millennium BC – 15th century AD). PhD thesis, The University of Edinburgh, UK. (10.7488/era/1960)

[38] Owen OA. 1993 Tuquoy, Westray, Orkney: a challenge for the future? In The Viking age in Caithness, Orkney and the North Atlantic (eds CE Batey, J Jesch, CD Morris), pp. 318-339. Edinburgh, Scotland: University Press.

[39] Owen OA. In preparation. Tuquoy: a Norse chieftain's farm in Westray, Orkney. Site monograph in preparation.

[40] Hillson S. 2005 Teeth, 2nd edn. Cambridge, UK: Cambridge University Press.

[41] Lawrence MJ, Brown RW. 1973 Mammals of Britain: their tracks, trails and signs, revised edn. London, UK: Blandford Press.

[42] Vigne JD. 1995 Détermination ostéologique des principaux éléments du squelette appendiculaire d'Arvicola, d'Eliomys, de Glis et de Rattus. Fiches d'ostéologie animale pour l'archeologie: Serie B: Mammifères, Editions APDCA **6**, 4-12.

[43] Ronniger M. 2009 Troian mice: a contribution to postcranial morphology of the rodent genera *Mus*, *Apodemus*, *Microtus*, *Cricetulus* and *Meriones*. BioArchaeologica **5**, 227-235.

[44] Fernández FJ, del Papa LM, Moreira GJ, Prates L, de Santis LJM. 2011 Small mammal remains recovered from two archaeological sites in the middle and lower Negro River valley (Late Holocene, Argentina): taphonomic issues and paleoenvironmental implications. Quat. Int. **245**, 136-147. (10.1016/j.quaint.2010.12.027)

[45] Dawson AB. 1925 The age order of epiphyseal union in the long bones of the albino rat. Anatomical Rec. **31**, 1-17.

[46] Adamczewska-Andrzejewska KA. 1967 Age reference model for *Apodemus flavicolis* (Melchior 1834). Ekologia Polska – Seria A **XV**, 787-790.

[47] Steiner HM. 1967 Untersuchungen über die Variabilität und Bionomie der Gattung Apodemus (Muridae, Mammalia) der Donau-Auen von Stockerau (Niederösterreich). Zeitschrift für wissenschaftliche Zoologie **177**, 1096.

[48] Lidicker Jr WZ. 1966 Ecological observations on a feral house mouse population declining to extinction. Ecol. Monogr. **36**, 27-50.

[49] Brothwell DR. 1981 The Pleistocene and Holocene archaeology of the house mouse and related species. Symp. Zool. Soc. Great Britain **47**, 1-13.

[50] Flowerdew JR, Tattersall FH. 2008 Apodemus sylvaticus. In Mammals of the British Isles, 4th edn. (eds S Harris, DW Yalden), pp. 125-137. Southampton, UK: The Mammal Society.

[51] Berry RJ, Tattersall FH, Hurst J. 2008 House mouse. In Mammals of the British Isles, 4th edn. (eds S Harris, DW Yalden), pp. 141-149. Southampton, UK: The Mammal Society.

[52] Rstudio Team. 2016 Rstudio: integrated development for R. Boston, MA: Rstudio, Inc. See www.rstudio.com

[53] Adler J. 2012 R in a nutshell: a desktop quick reference. Cambridge, UK: O'Reilly Media.

[54] Booth C, Booth J. 2005 Sillocks, skarfies & selkies: the fish, amphibians, reptiles, birds and mammals of Orkney. Hatston, TX: The Orcadian Ltd.

[55] White CM. 1994 Family Falconidae. In Handbook of birds of the world. Volume 2 New World vultures to guineafowl (eds J del Hoyo, A Elliott, J Sargatal), pp. 216-275. Barcelona, Spain: Lynx Edicions.

[56] Orta J. 1994 Common kestrel. In Handbook of birds of the world. Volume 2 New World vultures to guineafowl (eds J del Hoyo, A Elliott, J Sargatal), pp. 259-260. Barcelona, Spain: Lynx Edicions.

[57] Reynolds P. 1992 The impact of changes in land-use in Orkney, on the vole *Microtus arvalis orcadensis* and its avian predators. Unpublished PhD thesis, The University of Aberdeen, UK.

[58] Andrews P, Fernández-Jalvo Y. 2018 Seasonal variation in prey composition and digestion in small mammal predator assemblages. Int. J. Osteoarchaeol. **28**, 318-331. (10.1002/oa.2656)

[59] Weissbrod L, Dayan T, Kaufman D, Weinstein-Evron M. 2005 Micromammal taphonomy of el-Wad Terrance, Mount Carmel, Israel: distinguishing cultural from natural depositional agents in the Late Natufian. J. Archaeol. Sci. **32**, 1-17.

[60] Lyman RL. 1994 Vertebrate taphonomy. Cambridge, UK: Cambridge University Press.

[61] Whyte TR. 1991 Small-animal remains in archaeological pit features. In Beamers, bobwhites, and blue-points: tributes to the career of Paul W. Parmalee (eds JR Purdue, WE Klippel, BW Styles), pp. 163-176. Springfield, MA: Illinois State Museum Scientific Papers, Vol. 23.

[62] Millais JG. 1904 On a new British vole from Orkney Islands. The Zoologist **757**, 241-246.

[63] Churchfield S, Searle JB. 2008 Pygmy shrew. In Mammals of the British Isles, 4th edn (eds S Harris, DW Yalden), pp. 267-271. Southampton, UK: The Mammal Society.

[64] Churchfield S. 1990 The natural history of shrews. London, UK: Christopher Helm A&C Black.

[65] Morris C, Ballin Smith B. 1996 The Birsay Bay project. Vol. 2, Sites in Birsay village and on the Brough of Birsay, Orkney, (monograph series no. 2) . Durham, NC: Department of Archaeology, University of Durham.

[66] Berry RJ ed. 1981 Biology of the house mouse: Symposia of the Zoological Society of London 47. London, UK: Academic Press.

[67] Suzuki H et al. 2013 Evolutionary and dispersal history of Eurasian house mice *Mus musculus* clarified by more extensive geographic sampling of mitochondrial DNA. Heredity **111**, 375-390. 23820581 10.1038/hdy.2013.60 PMC3806020

[68] Nicholson RA, Barber P, Bond JM. 2005 New evidence for the date of introduction of the house mouse, *Mus musculus domesticus* Schwartz & Schwartz, and the field mouse, *Apodemus sylvaticus* (L.), to Shetland. Environ. Archaeol. **10**, 143-151.

[69] Guiry EJ, Gaulton BC. 2016 Inferring human behaviors from isotopic analyses of rat diet: a critical review and historical application. J. Archaeol. Method Theory **23**, 399-426. (10.1007/s10816-015-9248-9)

[70] Swift JA, Miller MJ, Kirch PV. 2017 Stable isotope analysis of Pacific rat (*Rattus exulans*) from archaeological sites in Mangareva (French Polynesia): the use of commensal species for understanding human activity and ecosystem change. Environ. Archaeol. **22**, 283-297. (10.1080/14614103.2016.1216933)

[71] Romaniuk AA, Troalen LG, Bendrey R, Herman JS, Owen O, Smith C. 2023 Supplementary material from “Pests or prey? Micromammal species within an ancient anthropic environment at the Norse settlement site of Tuquoy (Westray, Orkney)”. Figshare. (10.6084/m9.figshare.c.6486202) PMC10073909 37035288

